# Glucocerebrosidase mutations disrupt the lysosome and now the mitochondria

**DOI:** 10.1038/s41467-023-42107-7

**Published:** 2023-10-11

**Authors:** Andrés D. Klein, Tiago Fleming Outeiro

**Affiliations:** 1 Centro de Genética y Genómica, Facultad de Medicina, Clínica Alemana Universidad del Desarrollo, Santiago, 7780272 Chile; 2 Department of Experimental Neurodegeneration, Center for Biostructural Imaging of Neurodegeneration, University Medical Center Göttingen, Göttingen, Germany; 3 Max Planck Institute for Natural Sciences, Göttingen, Germany; 4 Translational and Clinical Research Institute, Faculty of Medical Sciences, Newcastle University, Framlington Place, Newcastle Upon Tyne, UK; 5 Scientific Employee with an Honorary Contract at Deutsches Zentrum für Neurodegenerative Erkrankungen (DZNE), Göttingen, Germany

**Keywords:** Parkinson's disease, Organelles, Molecular medicine

## Abstract

β-Glucocerebrosidase (GCase) mutations lead to glucosylceramide build-up in the lysosome, impacting α-synuclein aggregation and autophagy. Recently, Baden and colleagues found GCase in mitochondria, supporting mitochondrial complex I function and energy metabolism. We believe the newly described role of GCase in the mitochondria will inform new Parkinson’s and Gaucher’s disease therapeutics.

Parkinson’s disease (PD) is a common neurodegenerative condition known for typical motor symptoms associated with the loss of dopaminergic neurons in the substantia nigra. Early genetics studies on familial forms of PD implicated variants in genes related to intracellular trafficking and mitochondrial biology, including *SNCA* (α-synuclein; a-syn), *LRRK2*, *DJ-1*, *PINK1*, *PARKIN*, and others^1^. However, recent large-scale next-generation sequencing studies have identified variants in one allele of the β-glucocerebrosidase (GCase) encoding gene (*GBA1*) in 5–30% of PD cases, depending on their ancestry^1^. From a clinical perspective, PD patients harboring *GBA1* mutations typically present with early onset of the disease, experience more rapid progression of motor impairment and cognitive decline, and have reduced survival rates^2^, suggesting that GCase plays a central role in the pathophysiology.

Biallelic *GBA1* mutations cause Gaucher’s disease (GD), a rare monogenic lysosomal storage disorder (LSD). The lysosomal GCase enzyme breaks down glucosylceramide (GlcCer) into glucose and ceramide. Consequently, GD cells cannot metabolize GlcCer, leading to its intracellular accumulation. Clinically, GD symptoms vary widely, ranging from visceral disease (Type 1) to neuropathic manifestations (Types 2 and 3)^3^.

PD and GD neurons exhibit oxidative stress markers and alterations in mitochondrial complex I (CI)^4,5^. Additionally, both exhibit the accumulation of α-synuclein (a-syn)^6,7^. For unknown reasons dopaminergic neurons abundantly express GCase^8^. Loss-of-function variants in *GBA1* lead to lysosomal dysfunction, causing GlcCer accumulation which fuels a-syn aggregation in human induced pluripotent stem cell (iPSC) neurons^9,10^. Furthermore, a-syn inhibits the activity of mutated and non-mutated GCase in neurons and idiopathic PD brains, creating a bidirectional cycle that contributes to the disease process^11^ (Fig. 1). Dopaminergic iPSC neurons carrying *GBA1* mutations display elevated cytoplasmic calcium levels and impaired macroautophagy flux, but these phenotypes could be reversed after zinc-finger nuclease (ZFN)-mediated gene corrections^12^. Mutated GCase (mGCase) also impairs chaperone mediated autophagy (CMA), causing the accumulation of CMA substrates, including α-syn^10^.

**Fig. 1:**
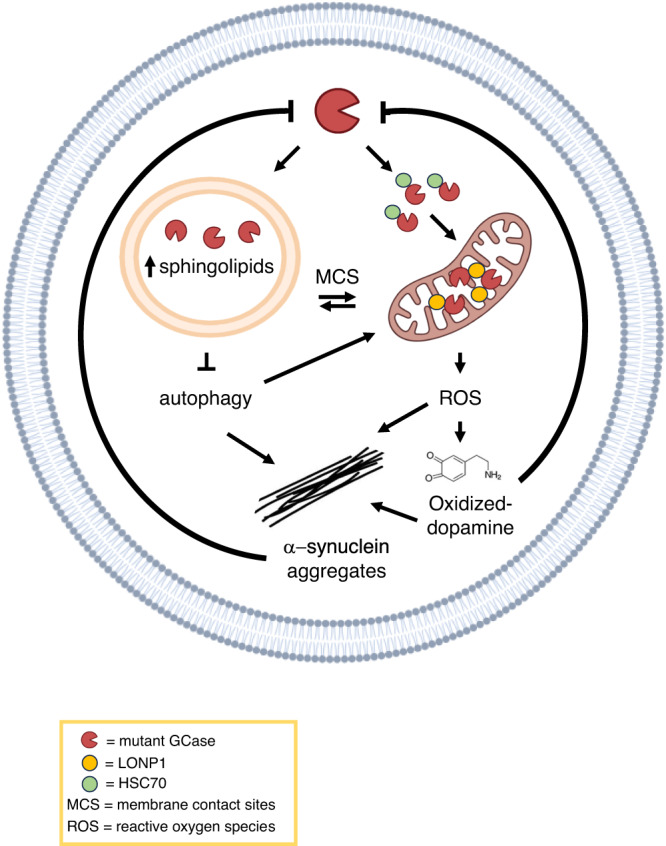
GCase exists in lysosomes and in mitochondria and plays a significant role in the pathological mechanisms of PD. Mutant GCase in lysosomes leads to elevated sphingolipid levels and hinders autophagic flux, resulting in the aggregation of a-syn into putatively toxic clusters. In turn, the accumulation of a-syn impairs GCase activity, initiating a detrimental cycle of cellular self-destruction. HSC70 imports GCase into mitochondria where it supports the proper functioning of mitochondrial complex I and maintains mitochondrial integrity. In this organelle, the protease LONP1 interacts with mutant GCase where it degrades it. Mutant GCase activity causes an increase in reactive oxygen species (ROS), leading to dopamine oxidation, increasing a-syn aggregation, and further impairment of GCase activity, creating a second pathological cycle. Additionally, autophagy deficiencies impair the elimination of a-syn and damaged mitochondria, leading to an additional rise in ROS levels. Furthermore, the lysosome and mitochondria are connected through membrane contact sites (MCS), facilitating the exchange of molecules, including toxic lipids and probably dysfunctional GCase. Black arrows indicate promotion of a mechanism and “T” indicates inhibition of the pathological loop.

Several lines of investigation suggested that GCase could play a role in mitochondria^13^. For instance, a mouse model of neuropathic GD showed dysfunctional and fragmented mitochondria with impaired respiration, reduced respiratory chain complex activities, and a decreased potential maintained by reversal of the ATP synthase^5^. Reduction in mitochondrial calcium uptake due to decreased levels of the mitochondrial calcium uniporter is also observed in *Gba1* deficient cells^14^. Furthermore, inhibition of GCase activity with Conduritol B-Epoxide and mitochondrial complex I (CI) with rotenone leads to similar results in terms of increased secretion of dopamine and serotonin metabolites to the extracellular medium^15^, suggesting that GCase and mitochondrial CI participate in a common pathway related to the dopamine/serotonin deficiency seen in PD.

However, a direct connection between GCase and mitochondrial CI was unclear. In a recent study published in *Nature Communications* by Baden and colleagues, the authors explored the interactome of wild type (WT) and mutant GCase (mGCase) proteins using a quantitative unbiased proteomics approach in HEK cells, iPSC-derived neurons and midbrain organoids^16^. Interestingly, 19 of the top 100 binding partners of WT-GCase were mitochondria-associated proteins. The authors validated the physical interaction of GCase with a cytosolic chaperone involved in protein folding prior to mitochondrial import (HSC70), a mitochondrial outer membrane (TOM70), inner membrane (TIM23 and ATP5B), and mitochondrial matrix (HSP60 and LONP1) proteins through immunoprecipitation^16^.

Analysis of the interactome of mGCase showed an enriched interaction with proteins involved in mitochondrial protein quality control, including LONP1, and a decreased interaction with TIMMDC1, the mitochondrial CI assembly factor. To demonstrate that both WT and mGCase can locate in mitochondria, the authors performed subcellular fractionation and super-resolution imaging. Using bioinformatics analysis, they predicted that GCase possesses an internal (non-canonical) mitochondrial targeting signal, which was experimentally validated. Baden and colleagues also showed that GCase, both WT and mutant, interact with HSC70, mediating their transport into mitochondria. Interestingly, LONP1 interacts with mGCase^16^, a mitochondrial protease that can degrade it and participates in a-syn aggregation. This study is consistent with our own work implicating another chaperonin protein, HSP10, as a protein that can be trapped by a-syn in the cytosol, thereby affecting mitochondrial function^17^.

So, what does GCase do in mitochondria? To answer this, the authors identified that mGCase showed reduced interactions with TIMMDC1 and NDUFA10, two proteins responsible for CI maintenance. Based on this observation, they hypothesized that inside mitochondria, GCase plays a role in ensuring proper mitochondrial CI function. To evaluate the higher order of CI integrity and its supercomplexes (also known as respirasomes), they utilized several complementary biochemical techniques. The amount of respirasomes was decreased in *GBA1* null and *GBA1* mutant neural progenitor cells and dopaminergic neurons compared to the WT protein, suggesting an unexpected role for GCase in the structural maintenance of CI.

The authors also investigated the impact of GCase on CI activity measured as the ratio of NADH oxidase/coenzyme Q reductase and oxygen consumption rates activities (OCR). Mutant GCase leads to decrease OCR and CI activity compared to WT GCase, but not in complexes II-IV, demonstrating a role for GCase in CI function^16^. Additionally, dysfunction of respiration caused by GCase deficiency leads to an increase in reactive oxygen species (ROS)^5,18^ which triggers dopamine oxidation in mitochondria in a non-enzymatic reaction. To close the cycle, dopamine adducts further inhibit GCase function, exacerbating the pathology^18^.

Finally, it is worth noting that mitochondria and lysosomes interact through specialized contact-sites^13^. Interestingly, PD-*GBA1* patient-derived dopaminergic neurons exhibit prolonged mitochondria-lysosome contacts due to defective modulation of the untethering protein TBC1D15, which mediates Rab7 GTP hydrolysis for contact untethering. Increasing GCase activity with S-181, a GCase small molecule chaperone, restored lysosome-mitochondria contact sites to normal^19^, suggesting a role for lysosome-mitochondria contact sites in PD pathology (Fig. 1).

## Concluding remarks and outlook

In conclusion, the mechanistic link between *GBA1* variants and PD may lie in the interplay between GCase functions in the lysosome and mitochondria. GCase mutations that affect function in the lysosome lead to the intracellular accumulation of glycolipids, disrupting normal lysosomal function and autophagy, and leading to a-syn accumulation. Additionally, a-syn buildup impairs GCase activity, creating a self-perpetuating cycle of lysosomal dysfunction and a-syn accumulation, which may trap mitochondrial proteins in the cytosol, exacerbating both mitochondrial and lysosomal dysfunction. This could be investigated using similar proteomics approaches to Baden and colleagues, attempting to assess protein interactions in native conditions. GCase is also imported into the mitochondria via HSC70, where it promotes the integrity and function of mitochondrial CI. GCase mutations increase its interaction with LONP1, degrading GCase in mitochondria and promoting a-syn aggregation. Thus, mGCase increases ROS in mitochondria, oxidizing dopamine. In turn, the accumulation of oxidized dopamine-adducts further impairs GCase activity, creating a second cycle of GCase dysfunction (Fig. 1).

The study by Baden et al.^16^ further supports developing and testing GCase-based therapeutics in addition to the modulation of the mitochondrial quality control system for diseases with GCase dysfunction. We expect to observe novel brain penetrant GCase therapeutics, which in combination with other ongoing efforts such as dopamine replacement or stem cell approaches, may lead to important to urgently needed disease modifying therapies.
